# Does Rectal Indomethacin Given for Prevention of Post-ERCP Pancreatitis Increase Bleeding After Biliary Endoscopic Sphincterotomy or Cardiovascular Mortality? Post Hoc Analysis Using Prospective Clinical Trial Data

**DOI:** 10.1097/MD.0000000000000159

**Published:** 2014-12-05

**Authors:** Árpád Patai, Norbert Solymosi, Árpád V. Patai

**Affiliations:** From the 1st Department of Medicine and Gastroenterology, Sopron Elizabeth Teaching Hospital, Sopron and Department of Gastroenterology and Internal Medicine, Markusovszky University Teaching Hospital, Szombathely (AP); Department of the Physics of Complex Systems, Eötvös Loránd University, Budapest (NS); and 2nd Department of Medicine, Semmelweis University, Budapest, Hungary (AVP).

## Abstract

Rectal indomethacin has been proven to be effective for prevention of post-ERCP pancreatitis (PEP) but its impact on bleeding after biliary sphincterotomy (BABES) and cardiovascular mortality has not been extensively studied. We aimed to assess the effect of indomethacin on the rate of BABES and short-term cardiovascular mortality, particularly in patients receiving antiplatelet therapy (APT).

In this double-blind, randomized, placebo-controlled, single-center study, 100 mg indomethacin or placebo was given within 1 hour before biliary endoscopic sphincterotomy to in-patients including those who are receiving APT (acetylsalicylic acid [ASA] and/or clopidogrel). Cardiovascular mortality and BABES were observed for 30 days.

Of 576 randomized patients (289 indomethacin, 287 placebo), 87 patients used 100 mg/day ASA and 29 patients took 75 mg/day clopidogrel, among them 5 patients were on dual APT. The ASA and clopidogrel taking patients were older than patients without APT (*P* < 0.001), but these groups were similar in other parameters. BABES occurred similarly in different subgroups: indomethacin (8.0%) vs placebo (9.4%) (*P* = 0.56), ASA (10.3%) vs non-ASA (8.4%) (*P* = 0.54), clopidogrel (6.9%) vs nonclopidogrel (8.8%) (*P* > 0.99). No BABES was observed among patients on dual APT. There was no difference in cardiovascular mortality between subgroups (*P* > 0.99).

Results indicate that single dose of 100 mg indomethacin does not increase BABES rate and cardiovascular mortality. This result is independent from administering antiplatelet agents.

## Abstract 2

Supplemental Digital Content is available in the text

## INTRODUCTION

Rectal indomethacin has been proven to be effective for prevention of post-ERCP pancreatitis (PEP).^1–4^ Similar to other nonsteroidal anti-inflammatory drugs (NSAIDs), indomethacin has platelet inhibiting function and can be considered to elevate the risk of bleeding after biliary endoscopic sphincterotomy (BABES). Antiplatelet agents (APA), which are widely used in cardiovascular patients for the prevention of stent or atherothrombosis could further increase the risk of BABES. Indomethacin has been reported not only to influence the activity of acetylsalicylic acid (ASA),^5^ but also to elevate the risk of cardiovascular events in APA takers.^6^

According to European,^7^ American,^8^ and British^9^ clinical practice guidelines, biliary endoscopic sphincterotomy (BES) is classified as a high-risk endoscopic procedure causing hemorrhage, and the use of ASA is suggested in those receiving APAs. However the guidelines lack recommendations for those patients who take APA regularly and receive indomethacin for PEP prophylaxis, and have to undergo urgent BES. In this prospective, randomized, double blind trial, study participants undergoing BES received 100 mg indomethacin for prophylaxis of PEP within 1 hour before ERCP. The aim of this work was to study BABES and cardiovascular mortality after indomethacin and analyze its effect in APA takers’ subgroups.

## METHODS

### Study Design

Between December 15, 2008, and January 13, 2013, a total of 576 patients with intact papilla underwent BES at the 1st Department of Medicine and Gastroenterology, Sopron Elizabeth Teaching Hospital. All patients were included in a randomized, prospective, double blind clinical trial to study the effect of rectally administered indomethacin for BABES. ASA 100 mg or clopidogrel 75 mg, if those were indicated, were not discontinued. This study was performed in accordance with the Declaration of Helsinki and the ICH Guidelines for Good Clinical Practice. The protocol was approved by the Medical Ethics Committee of Sopron Elisabeth Teaching Hospital (17-40/2008).

### Randomization and Masking

Indomethacin treatment allocation and concealment were conducted in a blinded fashion by an independent pharmacy staff in a separate building preparing suppositories of identical appearance that contained either 100 mg indomethacin or placebo. Block size of 20 was chosen to make sure participants were enrolled with a 1 : 1 ratio of the indomethacin group to the placebo group. Neither patients nor investigators were able to differentiate participant's treatment group.

### Study Protocol

A single dose of suppository was administered rectally within 1 hour before BES as peak concentration of indomethacin in suppository form is known to occur between 30 and 90 minutes after insertion with an elimination half-life of 2 hours.^1,10^ Each patient was observed in the hospital at least for 24 hours and heart rate, blood pressure, and blood counts were measured 24 hours after BES. Patients received at least 2000 mL IV fluids. The protocol described to break the code of indomethacin at anaphylaxis or at discretion of treating team. Platelet transfusion was available, if it was necessary.

### Patients

**FIGURE 1:**
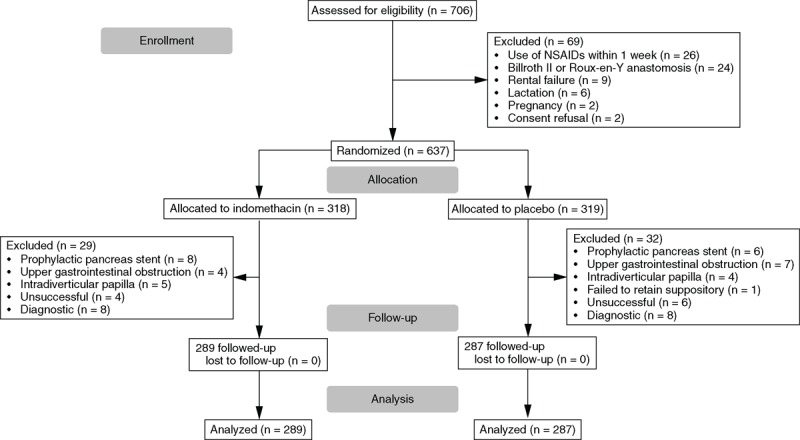
Patient enrollment and outcomes.

ERCP with proposed biliary therapy was performed in unselected, consecutive in-hospital patients with naïve papilla following routine admission; history, physical examination, abdominal ultrasonography, and laboratory tests (Figure 1). Exclusion criteria included age under 18 years, pregnancy or lactation, upper gastrointestinal obstruction, history of Billroth II or Roux-en-Y anastomosis, history of allergy to indomethacin or contrast medium, serum creatinine level above 1.41 mg/dL (125 μmol/L), international normalized ratio (INR) above 1.5 (if it was necessary, vitamin K and/or fresh frozen plasma was administered to decrease INR), platelet count <50×10^9^/L, the use of NSAIDs within 1 week before admission and refusal to participate in the study. Temporary holding of antiplatelet therapy (APT) was considered carefully in each patient before ERCP. If APAs were not necessary, they were discontinued at least 7 days before ERCP. If ASA and/or clopidogrel therapy could not be safely interrupted before BES, ERCP was performed during maintained APT. Patients with sphincterotomy of the minor papilla, previous severe PEP, or papillectomy were excluded; in these cases, insertion of prophylactic pancreatic stent was performed. All patients gave written informed consent before enrollment.

### ERCP

All ERCP procedures were performed by 1 endoscopy expert (AP) with standard therapeutic duodenoscope (Fuji EPX2200 processor, ED 250XT8, Fujifilm, Tokyo, Japan) in short loop position. The patients received topical pharyngeal anesthesia (1% lidocaine) and intravenous administration of midazolam (5 to 10 mg) and/or atropine (0.5 mg) and/or pethidine 50 to 100 mg for moderate sedation. Buthylscopolamine (Buscopan, Boehringer Ingelheim GmbH, Ingelheim am Rhein, Germany) was used as smooth muscle relaxant according to the endoscopist's decision. Last dose of low molecular weight heparin (LMWH) was given at least 12 hours before BES and ERCP was performed at INR <1.5. Heart rate and oxygen saturation were monitored; nasal cannula oxygen was given if necessary. Air was used for luminal insufflation during ERCP. The cannulation of common biliary duct was performed by papillotome (with double lumen, tapered nose, cutting length of 30 mm, Medwork PAP1-CF-20–35-OL, Medwork GmbH, Höchstadt/Aisch, Germany) and hydrophilic guide wire (with a diameter of 0.035-in., Jagwire, Boston Scientific Corp., Natick, MA) if necessary. Contrast medium amidotrizoate (Peritrast 600 mg/mL, Dr. Franz Köhler Chemie GmbH, Bensheim, Germany) was diluted to 50% in distilled water and injected manually. Zipper cut was planned to be avoided in every BES. During our previous 6000 ERCPs, we experienced moderate and very rarely serious BABES without mortality; however, moderate and serious PEPs (among them necrotizing PEP leading to death) occurred much more frequently. For this reason, in 2007 and 2008 when we planned this trial, accepting opinion of others,^11–15^ we preferred prevention of PEP and decided to apply pure-cut current (45W) (Surgistat, Valleylab, Covidien, Boulder, CO) in all endoscopic sphincterotomies.

### BABES

Cases with BABES were divided into 2 categories: immediate (intraprocedural) and delayed (postprocedural) bleeding. As in our praxis appearance of some oozing after BES is frequent with no further consequences, it was not considered as a bleeding event. Intraprocedural bleeding was diagnosed in cases in which hemostasis (electrocoagulation or injection of epinephrine) was necessary during biliary endoscopy. This procedure was indicated in 2 situations: hemorrhage was intensive according to the judgment of our expert, or bleeding did not stop 3 min after BES or the last endoscopic therapeutic maneuver. Postprocedural bleeding was defined in patients with clinical signs of bleeding or if the decrease of hemoglobin level (Hgb) was greater than 20 g/L within 24 hours but after completion of BES and subsequent upper gastrointestinal endoscopy did not show other source of bleeding. Bleeding was graded by consensus criteria^16^: mild bleeding was defined as clinical (not just endoscopic) evidence of bleeding, Hgb drop was <30 g/L, and no transfusion was necessary; moderate bleeding was defined as Hgb drop ≥30 g/L or the need for transfusion was no >4 units and angiography or surgery was not necessary; serious bleeding was defined when the need for transfusion was 5 or more units or angiographic or surgical interventions were necessary. Each patient was monitored for at least 30 days. Every discharged patient was informed about the signs of gastrointestinal hemorrhage and was asked to return if they noticed any signs of bleeding. Patients were checked within 6 weeks after BES and were asked about symptoms of hemorrhage. In case of the death of a patient, autopsy was planned in every case.

### Statistical Analysis

This work is a subsequent analysis of our PEP study.^4^ In this work patients without BES were excluded, but the current cohort included 63 patients with acute biliary pancreatitis originally excluded from the PEP study (Figure 1). For the univariate analysis of outcome (PEP) two-tailed Fisher exact test was used.^17^ Student's *t*-test was used to compare groups with continuous variables. Multivariate analysis was performed by logistic regression. A *P* value of <0.05 was considered significant. All statistical analyses were performed by using the R-environment.^18^

## RESULTS

### Patients’ Disposition and Baseline Characteristics

A total of 637 patients with proposed biliary endoscopic therapy were initially randomized into this study (Figure 1). Sixty-one patients were excluded after allocation: in 20 patients, the major papilla could not be reached because of upper gastrointestinal obstruction and its intradiverticular position in 10 patients the cannulation of bile duct was unsuccessful. In 16 patients, endoscopic therapy was not necessary; therefore, BES was not performed. Fourteen patients received pancreatic stent according to protocol. An additional patient was excluded from the placebo group because of inability to retain the suppository in the rectum. Finally, 576 patients were analyzed. Zipper cut could be avoided in all BES cases.

In our earlier study indomethacin decreased PEP rate from 13.8% to 6.7% (*P* < 0.007).^4^ Two-hundred eighty-nine patients in the indomethacin group and 287 patients in the placebo group did not differ from each other in mean age, age ≤50 years, gender, average platelet count, papillary stenosis, and endoscopic procedures (frequency of precut, lithotripsy, biliary stent implantation), only cholangitis occurred more frequently in the indomethacin group (*P* < 0.01) (Table 1, Supplementary Table 1, http://links.lww.com/MD/A76). In our trial, 87 patients used 100 mg/day of ASA and 29 patients regularly received 75 mg/day of clopidogrel, 5 patients had dual APT. Patients on ASA and clopidogrel were significantly older (*P* < 0.001) than those not taking APAs, and among patients not taking ASA there were significantly more females (*P* < 0.05). Patients on APT did not differ from each other in platelet count and endoscopic procedures (Table 1).

**TABLE 1:**
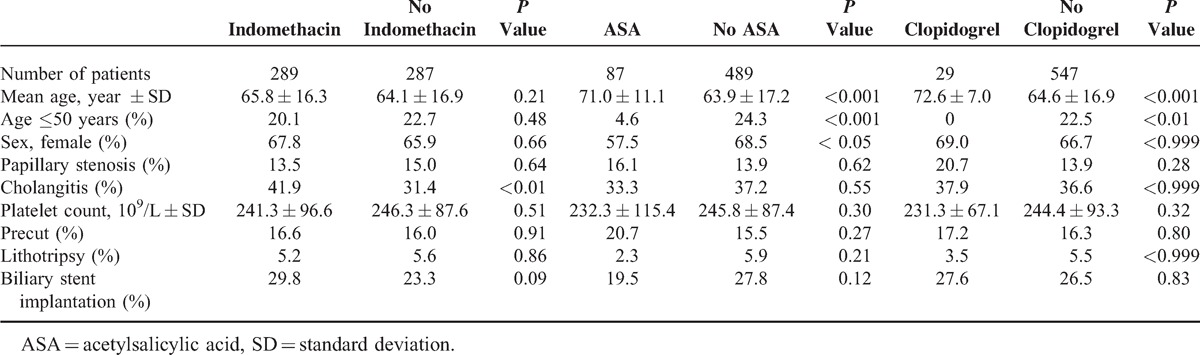
Antiplatelet Therapy, Demographical, and Clinical Characteristics of Study Patients

### BABES

BABES occurred in 50 patients (8.7%) with intraprocedural bleeding in 17 patients (3%) and postprocedural bleeding in 36 (6.3%) patients. In 3 cases, postprocedural hemorrhage recurred after initially successful endoscopic therapy of intraprocedural bleeding. No extraluminal hemorrhage was detected. Types of bleeding were compared based on prior use of indomethacin, ASA, or clopidogrel (Table 2, Supplementary Table 2, http://links.lww.com/MD/A76). Our data showed no significant impact of the use of APAs on subsequent rates of intraprocedural, postprocedural, and total bleeding. Of note, 5 patients (among them 2 patients receiving indomethacin) treated with both clopidogrel and ASA had no bleeding complications. When intraprocedural bleeding occurred, we immediately switched from pure-cutting current to coagulation in 13 cases, and the bleeding stopped. In another 4 patients, coagulation was applied after 3 minutes. In 1 of these 4 cases, coagulation was insufficient, therefore, 2 mL diluted epinephrine was injected. All the bleedings were stopped at the end of ERCP. Postprocedural bleeding was detected from 3 hours to 19 days after BES (in 30 out of 36 patients with postprocedural bleeding BABES was observed in the first 24 hours), but endoscopic therapy was only necessary in 5 cases. Injection of diluted epinephrine was effective in 4 patients, in 1 patient heat probe unit was applied after injection of diluted epinephrine. Two of these patients were on clopidogrel: 1 patient used ASA, and 2 patients received indomethacin before BES. No patient required surgery; however, 1 patient had to receive 2 units of red blood cells, and she did not take any APAs.

**TABLE 2:**
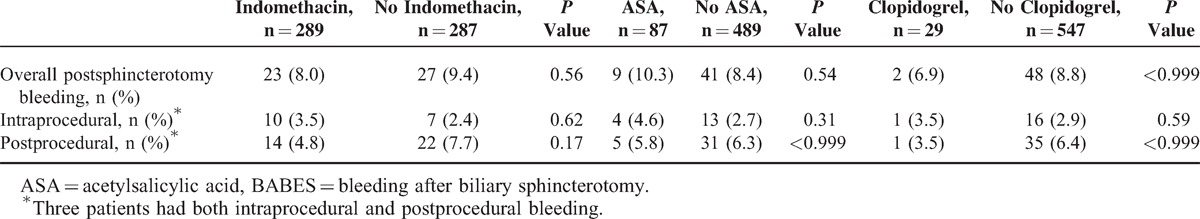
BABES in Patients Treated With Indomethacin, ASA, and Clopidogrel

On 30-day follow-up, we did not detect mortality related to BABES (Supplementary Table 3, http://links.lww.com/MD/A76). One patient died from PEP (0.2%), she did not take indomethacin, ASA, or clopidogrel. Nine patients (1.6%) died due to causes independent of the procedure; in the indomethacin group 1 ASA user died due to cardiac failure, 1 patient died due to complications of cholecystectomy, and 1 patient died due to malignant disease, whereas in the placebo group 1 patient died due to myocardial infarction (she did not take ASA or clopidogrel) and 3 patients died due to malignant disease. One patient in the indomethacin and 1 patient in the placebo group died due to severe necrotizing biliary pancreatitis despite successful BES and biliary stone extraction. All deceased patients underwent autopsy, no signs of bleeding was detected. Cardiovascular mortality in groups of different APAs users did not differ from each other.

Patients with and without bleeding did not differ from each other in age, sex, endoscopic therapy, and APA use; however, patients with BABES had lower platelet count (*P* = 0.01) (Table 3). Multivariate analysis did not show any risk for bleeding regarding demographical data, endoscopic procedures, and different APAs (Table 4). BABES was graded according to consensus criteria.^16^ Forty-three patients (86%) had mild bleeding and 7 patients (14%) had moderate bleeding. We did not detect any serious bleeding. Grade of hemorrhage did not differ from each other according to different APAs. We investigated the interaction of indomethacin and ASA (Table 5). There was no significant difference concerning BABES.

**TABLE 3:**
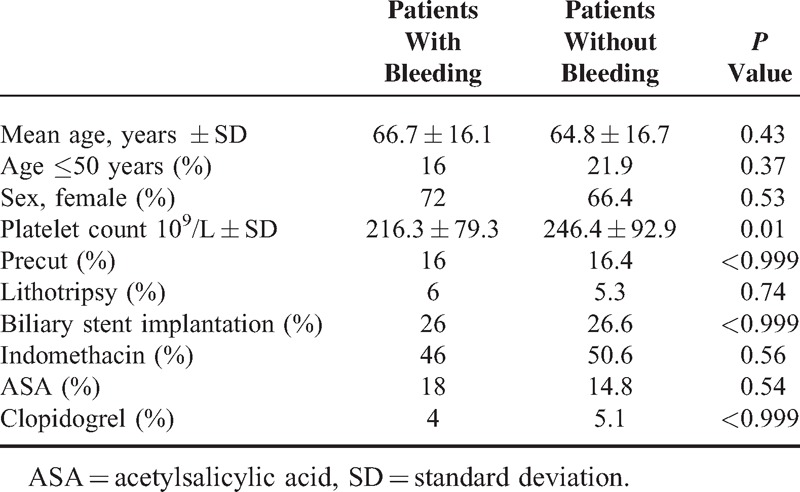
Comparison of Patients With and Without Bleeding

**TABLE 4:**
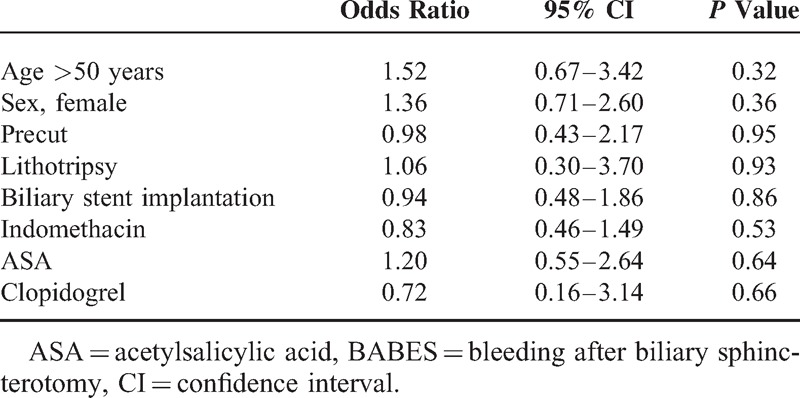
Multivariate Analysis Identifying Risk Factors for BABES

**TABLE 5:**
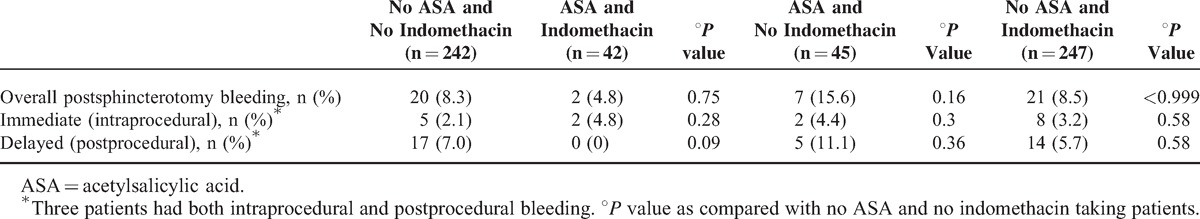
Post-Sphincterotomy Bleeding in ASA and Indomethacin Users

## DISCUSSION

According to our knowledge, this is the first prospective randomized, double blind trial for analyzing the occurrence of BABES in patients using single dose of indomethacin and additional ASA (n = 87) and clopidogrel (n = 29). Our results indicate that there is no difference in the risk of BABES with regard to the prior use of indomethacin and holding of ASA or clopidogrel even though patients taking ASA and clopidogrel in our cohort were significantly older than those without APT.

Guidelines suggest holding the administration of clopidogrel before BES and continuing ASA only in patients treated by dual APT, if it is not contraindicated.^7–9^ It has been reported that ASA and NSAIDs do not increase the risk for bleeding after colonic polypectomy,19 a procedure with similar bleeding risk to BES.^19^ Publications for BABES in patients using ASA or NSAIDs are controversial. According to the only prospective study related to this subject, BABES was independent from the use of ASA and NSAIDs within 3 days preceding endoscopic intervention.^20^ In a retrospective case-control study, BABES was significantly more frequent regardless whether patients stopped taking ASA 1 week before BES or remained on ASA throughout BES.^21^ However, this result seems to be controversial as evidence indicates that platelet aggregation is normalized 7 days after stopping ASA treatment.^22^ In addition, this study^21^ was criticized by Hussain and coworkers^18^ for the definition of BABES and statistical analysis. In Hussain's retrospective case-control study, ASA and NSAID did not increase risk for BABES.^23^

The incidence of BABES can range widely from 1.1% to 48% depending on the rate of therapeutic ERCP and on the definition of BABES.^16,20,24–29^ As far as we are concerned, our definition is able to register all bleeding events (excluding spontaneous resolution) and, therefore, we can obtain exact information of incidence of BABES to compare the effect of APAs. Frequency of BABES observed in our study was 8.7%, corroborating the findings of a study, in which the rate of BABES was 10.4% if epinephrine injection was applied when bleeding did not stop 2 minutes following BES.^30^ According to literature recommended, waiting time for spontaneous stopping of hemorrhage before initializing endoscopic hemostasis varies between 3 minutes^31^ and 5 minutes^32^. In our study, this time was defined as 3 minute. Mortality of BABES ranges from 0.3%^16^ to 3.54%,^28^ although in our cohort we detected no mortality due to BABES. We classified BABES as mild in 86% of patients, which is similar to the rates found by Kim et al^30^ (79.4%) and accepted as standard by the ASGE (70%),^27^ but considerably higher than found by Freeman et al^20^ (29.2%). We observed moderate bleeding in 14% of our patients with BABES, similar to ASGE standards (18%),^27^ and to Kim et al^30^ findings (16.2%). Severe bleeding did not occur in our patients. We detected intraprocedural bleeding in 3% of BES, ranging from 1% to 14%,^20,32^ whereas 6.3% postprocedural bleeding, which is similar to 10.3 and 4.2% in earlier studies.^30,31^

Risk factors for bleeding include precut, papillary stenosis, acute cholangitis, coagulopathy, and anticoagulant therapy within 72 hours of sphincterotomy.^20,25–28,33^ Patients taking different APAs did not differ from each other in the above categories. However, the frequency of acute cholangitis was higher in the indomethacin group compared with the placebo group. In spite of our expectations that more frequent acute cholangitis can result in more BABES in the indomethacin group than in the placebo group, BABES occurrence was similar in the indomethacin and in the placebo group. In our study, platelet number was significantly lower in bleeding patients, but APAs did not significantly influence bleeding. Moreover, BES was performed only if the INR did not exceed 1.5. Precut was performed relatively frequently, which was a consequence of our cannulation strategy: early precut could improve the success of ERCP and decrease PEP rate.^4,34^ Although the use of NSAIDs, such as indomethacin, has been reported to influence the activity of ASA,^5^ and increase the risk of cardiovascular events,^6^ we made no such observations in this cohort. In fact, none of the 5 patients who remained on both ASA and clopidogrel suffered from BABES similarly to observations in a recent study.^35^ These data may deserve further investigations on larger patient cohorts.

Our study has several limitations. This was a single-center study with only the administration of indomethacin being randomized into a double-blind study while the use of ASA and clopidogrel use was examined in a prospective, nonrandomized, open series. We applied pure-cut current, although recent publications suggest applying blended current at first.^36,37^ Further multicenter trials are required to clarify the effect of APAs on BABES.

In conclusion, results of our prospective study indicate that single dose of indomethacin does not increase short-term cardiovascular mortality and this result is similar in APA takers and non-APA takers. Moreover, we provide evidence that the use of indomethacin for the prevention of PEP has no impact on the rates of intraprocedural and postprocedural bleeding following biliary sphincterotomy.
